# Next-generation sequencing of mixed genomic DNA allows efficient assembly of rearranged mitochondrial genomes in *Amolops chunganensis* and* Quasipaa boulengeri*

**DOI:** 10.7717/peerj.2786

**Published:** 2016-12-15

**Authors:** Siqi Yuan, Yun Xia, Yuchi Zheng, Xiaomao Zeng

**Affiliations:** 1 Department of Herpetology, Chengdu Institute of Biology, Chinese Academy of Sciences, Chengdu, Sichuan, China; 2 University of Chinese Academy of Sciences, Beijing, China

**Keywords:** Gene rearrangement, WANCY, Illumina sequencing, Neobatrachian, Mitogenome

## Abstract

Recent improvements in next-generation sequencing (NGS) technologies can facilitate the obtainment of mitochondrial genomes. However, it is not clear whether NGS could be effectively used to reconstruct the mitogenome with high gene rearrangement. These high rearrangements would cause amplification failure, and/or assembly and alignment errors. Here, we choose two frogs with rearranged gene order, *Amolops chunganensis* and *Quasipaa boulengeri*, to test whether gene rearrangements affect the mitogenome assembly and alignment by using NGS. The mitogenomes with gene rearrangements are sequenced through Illumina MiSeq genomic sequencing and assembled effectively by Trinity v2.1.0 and SOAPdenovo2. Gene order and contents in the mitogenome of* A. chunganensis* and *Q. boulengeri* are typical neobatrachian pattern except for rearrangements at the position of “WANCY” tRNA genes cluster. Further, the mitogenome of *Q. boulengeri* is characterized with a tandem duplication of *trnM*. Moreover, we utilize 13 protein-coding genes of *A. chunganensis*, *Q. boulengeri* and other neobatrachians to reconstruct the phylogenetic tree for evaluating mitochondrial sequence authenticity of *A. chunganensis* and *Q. boulengeri*. In this work, we provide nearly complete mitochondrial genomes of *A. chunganensis* and *Q. boulengeri*.

## Introduction

In metazoan mitochondrial genomes, the organization is usually conserved (Boore, 1999). The typical mitogenome of metazoans contains two ribosomal RNAs, 22 transfer RNAs (tRNAs), 13 protein-coding genes (PCGs) and non-genic regions. Because of maternal inheritance features and other characteristics of mitogenome (i.e., relatively conserved gene content and organization, rapid mutation rate, and limited recombination), mitochondrial DNA (mtDNA) is a valuable and popular molecular marker (Xia et al., 2014; Hahn, Bachmann & Chevreux, 2013; Zhang et al., 2013). It has been extensively applied in population genetics, evolutionary biology, phylogeography, as well as phylogenetic relationships at multiple taxonomic levels (Hahn, Bachmann & Chevreux, 2013; Zhang et al., 2013; Lindqvist et al., 2010). However, cases of mitochondrial reorganization (e.g., gene rearrangements, gene duplication and loss) are found in many lineages, even in closely-related species (Xia et al., 2014; Kurabayashi et al., 2010; Dowton et al., 2009). The rearrangements would cause amplification, assembly and alignment errors or failure.

The traditional protocols to produce the mitochondrial genomes have relied on performing either standard/long PCR or cloning, followed by a series of Sanger sequencing (Moreira, Furtado & Parente, 2015; Gan, Schultz & Austin, 2014; Poulsen et al., 2013). The classic method is a time consuming and resource demanding task (Hahn, Bachmann & Chevreux, 2013). Recently, the developments of next generation sequencing (NGS) have brought about PCR-free high-throughput sequencing to recover the whole mtDNA genomes (Machado, Lyra & Grant, 2015; Gan, Schultz & Austin, 2014; Hahn, Bachmann & Chevreux, 2013; Jex et al., 2010). The NGS method overcomes some of the current challenges to isolate mtDNA and the biases introduced by PCR (Smith, 2016; Dames, Eilbeck & Mao, 2015; Machado, Lyra & Grant, 2015; Tang et al., 2014; Hahn, Bachmann & Chevreux, 2013).

Numerous applications for rapidly assembling mitogenomes directly from NGS have been proposed (Cong & Grishin, 2016; Lounsberry et al., 2015; Cameron, 2014; Gan, Schultz & Austin, 2014). Based on high-throughput sequencing technologies, Tang et al. (2014) developed a novel multiplex sequencing and assembly pipeline for rapid and accurate reconstruction of full mitogenome from pooled *Drosophila* without DNA enrichment or amplification. The fast recovery, assembly, and annotation of mitogenome from genomic sequencing have been applied in butterflies and moths (Cong & Grishin, 2016), crayfish (Gan, Schultz & Austin, 2014), monogenean ectoparasitic flat-worms (Hahn, Bachmann & Chevreux, 2013), giant intestinal fluke (*Fasciolopsis buski*; Biswal et al., 2013) and Ascidian species (Rubinstein et al., 2013). In addition, RNA-seq and ultraconserved elements (UCE) sequencing are also excellent source to assemble mitochondrial genomes (Machado, Lyra & Grant, 2015; Moreira, Furtado & Parente, 2015; Raposo do Amaral et al., 2015).

The recent availability of complete mitogenomes and the partial sequences of “hotspots” of mtDNA rearrangements revealed a surprising array of gene organization in anurans, especially in neobatrachians (Xia et al., 2014; Kurabayashi et al., 2010; Ren et al., 2009). Gene order rearrangement involving the coding gene ND5 is observed in Dicroglossidae (*Euphlyctis*, *Hoplobatrachus*, *Fejervarya*; Alam et al., 2010; Ren et al., 2009; Liu, Wang & Su, 2005), Mantellidae (*Mantella*; Kurabayashi et al., 2008; Kurabayashi et al., 2006) and Rhacophoridae (*Rhacophorus*, *Buergeria*; Huang et al., 2016; Sano et al., 2005; Sano et al., 2004). Two copies of *trnM* genes are found in Dicroglossidae (e.g., *Feirana* and *Quasipaa*; Chen et al., 2015; Shan et al., 2014; Ren et al., 2009; Liu, Wang & Su, 2005) and Mantellidae (e.g., *Mantella*; Kurabayashi et al., 2008; Kurabayashi et al., 2006). The tRNA gene cluster, “WANCY,” is a hotspot of gene rearrangement in Neobatrachia, such as Ranidae (Xia et al., 2014; Kurabayashi et al., 2010), Dicroglossidae (Shan et al., 2014; Zhou et al., 2009) and Mantellidae (Kurabayashi et al., 2008; Kurabayashi et al., 2006). However, these rearranged sequences have been approached using the conventional procedure of combining long-range PCR with subsequent primer walking. Although high-quality mitochondrial genomes have been obtained from NGS for anurans (Machado, Lyra & Grant, 2015), more cases are needed to confirm the effectiveness of this protocol, especially for species with gene order rearrangement.

Some frogs with gene order rearrangements provide the opportunity to test the validity of mitogenome assembly by NGS. Gene order reorganization was reported in the “WANCY” gene cluster of some *Amolops* mitogenomes (Xia et al., 2014; Xue et al., 2015; Zhang, Xia & Zeng, 2016; Shan et al., 2016). The “WANCY” gene cluster was also rearranged in the spiny-bellied frog, *Quasipaa boulengeri* (Shan et al., 2014). Furthermore, the intraspecific diversity of gene rearrangements, which contains four kinds of rearrangements, within *Q. boulengeri* has been identified in 290 samples from 28 populations (Xia et al., 2016). The discovery of intermediate states and alternative losses types in the spiny-bellied frog provided direct evidence of tandem duplication and random loss model for mitochondrial gene rearrangements. However, such partial duplications and deletions of mtDNA fragments would cause inconvenience to assemble mitogenomes by NGS.

In this study, we choose two frogs with gene rearrangements, *Amolops chunganensis* and *Quasipaa boulengeri*, to test whether NGS could effectively obtain the mitogenome with high gene rearrangement. The mitogenomes are assembled and annotated from next generation sequencing reads through Illumina MiSeq genomic sequencing. The nearly-complete mitochondrial DNA sequence of *A. chunganensis* and *Q. boulengeri* were recovered, and compared with other *Amolops* and *Quasipaa* species, respectively. In order to evaluate mitochondrial sequence authenticity of *A. chunganensis* and *Q. boulengeri*, we performed a phylogenetic analysis. The phylogenetic tree was constructed by using Bayesian Inference (BI) and Maximum Likelihood (ML) methods for the two newly obtained and other published neobatrachian mitogenomes from GenBank.

## Materials and Methods

### Library preparation and Illumina sequencing

The sample of *Amolops chunganensis* was collected in Gansu Province, China (Voucher No. XM5526, ♀); and *Quasipaa boulengeri* was collected in Sichuan Province, China (Voucher No. XM3632, ♀). Chengdu Institute of Biology issued permit number CIB#2014-36 and CIB#2014-110 for field work. All work with animals was conducted according to relevant national and international guide-lines on the Protection of Wildlife. All animal care and experimental procedures were approved by the Animal Care and Use Committee (Permit Number: CIB-20121220A), Chengdu Institute of Biology, Chinese Academy of Sciences.

Genomic DNA was extracted from muscle tissue through SDS-proteinase K/phenol-chloroform protocols. DNA samples were shipped to Novogene Bioinformatics Technology (Beijing, China) for library construction and sequencing on Illumina MiSeq platform with 300 bp paired-end reads (PE300). Briefly, the paired-end library was performed with TruSeq kit (insert size ∼500 bp), following the protocols of Illumina DNA sample preparation. These libraries were pooled together to sequence on Illumina MiSeq platform at the Novogene Bioinformatics Technology (Beijing, China). Sequence files for genomic DNA from *A. chunganensis* and *Q. boulengeri* were generated in FASTQ format (sequence read and quality information in Phred format). The raw sequencing data were deposited in the National Center for Biotechnology Information (NCBI) Sequence Read Archive (SRA, http://www.ncbi.nlm.nih.gov/Traces/sra) under accession numbers SRR3929655 and SRR3929656 for *Q. boulengeri* and *A. chunganensis*, respectively.

### Mitogenome assembly

Contaminant sequences were first removed. Then, low-quality regions (Phred quality score < 20) and sequence adapters in the libraries were trimmed by using Trimmomatic (Bolger, Lohse & Usadel, 2014). *De novo* assembly of clean reads for each datasets were performed using SOAPdenovo2 (Luo et al., 2012) and Trinity v2.1.0 (Haas et al., 2013). Reads preprocess and assembly parameters followed the individual program guidelines. For SOAPdenovo2, we used 7 k-mer sizes between 50 and 80 with a step size of 5 and manually modified the assembly parameter values. Trinity assembler was used with the inchworm k-mer method, following authors’ recommendations. For each assembly, we monitored the change of total number of contigs and N50 size over the assessed parameter range.

After assembly, we used the published mitogenomes of *Amolops* as queries against the contigs dataset of *A. chunganensis*. *Amolops loloensis* (GeneBank No. KT750963) and *A. mantzorum* (KJ546429) were used as queries for the reference mitogenome. The reference mitogenomes were BLASTed against assembly using BLASTn (BLAST + v2.2.30) to search for contigs with mitochondrial protein-coding and RNA genes. For *Q. boulengeri*, the same strategy has been performed. *Quasipaa boulengeri* (KC686711) and *Q. spinosa* (NC_013270) were chose as reference mitogenomes.

### Mitogenome annotation and analysis

The mitogenome sequence was annotated using both tRNAscan-SE v.1.21 (http://lowelab.ucsc.edu/tRNAscan-SE; Schattner, Brooks & Lowe, 2005; Lowe & Eddy, 1997) and the MITOS web server (http://mitos.bioinf.uni-leipzig.de/index.py; Bernt et al., 2013). These programs also used to predict the potential cloverleaf secondary structures of tRNAs genes. The sequences of candidate tRNAs were recognized and aligned with homologous tRNAs genes. Thirteen PCGs of the two mitogenomes were identified by comparing inferred open reading frames with published *Amolops* and *Quasipaa* species. Both translation of open reading frames and annotation of the N- and C- terminal ends of each PCG were done in MEGA v6.06 (Tamura et al., 2013). In addition, the boundaries of the *rrnL* and *rrnS* genes were predicted by the flanking tRNA genes.

### Phylogenetic analysis

To validate the phylogenetic relationship of *A. chunganensis* and *Q. boulengeri* among neobatrachians, we aligned 46 mitogenomes of neobatrachians to confirm the phylogenetic relationships, and selected *Pelobates cultripes* (NC_008144), *Xenopus laevis* (NC_001573) and *X. tropicalis* (NC_006839) as outgroup taxa. Details of the GenBank accession numbers of anuran mitochondrial genomes analyzed were listed in Table S1. All sequences were edited manually in BioEdit Sequence Alignment Editor v.7.0.5 (Hall, 1999) and MEGA v.6.06 (Tamura et al., 2013) with default parameters. The alignments of 13 PCGs were determined in software MEGA v.6.06, with the default settings (align codons). The PCGs were translated to amino acids sequences and were manually concatenated all sequences into a single nucleotide dataset (total 11,502 bp).

We constructed the phylogenies with the concatenated dataset using BI and ML methods, which were conducted by MrBayes v.3.1 (Ronquist & Huelsenbeck, 2003; Huelsenbeck & Ronquist, 2001) and RAxML v.8.2.x (Stamatakis, 2014), respectively. Nucleotide substitution model selection was estimated under Bayesian Information Criterion (BIC) scores in jModeltest v.0.85 (Posada, 2008), and model GTR + I + G was selected as the best-fitting model for BI and ML analyses. BI partitioning analysis followed the programs with calculating a majority rule consensus tree with 10,000,000 generations of Markov chain Monte Carlo (MCMC), with frequency of tree sampling every 1,000 generations and the first 25,000 trees discarding as burn-in, and starting from a random tree. After performing two independent runs, the output trees were combined to estimate the Bayesian posterior probabilities (BPP) in 50% majority rule for each node. For ML analysis, program RAxML were performed with model GTRGAMMA under the similar partitioning parameters as BI analysis, and with 1,000 bootstrap replicates to calculate the bootstrap (BS) of the topology. In addition, the significant nodes’ supports were considered with 95% BPP (Felsenstein, 2004) and 75% BS (Hillis & Bull, 1993) in BI and ML analysis, respectively.

## Results

### Sequencing data from Illumina MiSeq

A total of 15.29 million raw PE reads were produced on Illumina MiSeq (PE300) systems (4.9 Gb and 4.2 Gb raw data for *Q. boulengeri* and *A. chunganensis*, respectively). After removal of adaptors and low-quality reads, the clean reads were used for the subsequent assembling. All assembly statistics of each assembler were shown in Table 1. Although total numbers of assembled contigs in Trinity were less than SOAPdenovo2, the Trinity assembler collected larger N50 and longer contigs (Table 1). More than two hundred contigs were aligned to reference mitogenome for each K-mer assembly of SOAP denovo2; however, most of them less than 500 bp. Further, few gaps existed when such contigs were used to assemble for the final mitogenome construction. By contrast, the best aligned contigs to reference mitogenome in Trinity are longer than 16 kb without gaps. It implies that Trinity is better than SOAPdenovo2 to recover mitogenome for low coverage metagenomic skimming data.

**Table 1:** Statistics and assembled result of SOAP denovo2 and Trinity.

Species	Assembly method	Total number of contigs	Total number of length	N50	Max contig length
*Quasipaa boulengeri*	K50^a^	6,341,679	1,860,878,175	300	2,615
	K55^a^	6,086,855	1,875,157,178	314	2,880
	K60^a^	5,715,340	1,870,183,020	332	3,183
	K65^a^	5,460,129	1,843,817,246	338	2,817
	K70^a^	5,122,048	1,779,429,007	332	2,980
	K75^a^	4,926,588	1,723,696,265	318	2,779
	K80^a^	4,686,214	1,630,358,728	300	2,830
	Trinity	1,555,983	755,862,296	526	16,672
*Amolops chunganensis*	K50^a^	5,072,290	1,417,075,271	300	2,537
	K55^a^	4,955,300	1,436,818,547	300	2,741
	K60^a^	4,746,605	1,436,171,325	300	2,416
	K65^a^	4,549,311	1,407,207,578	300	2,513
	K70^a^	4,301,826	1,350,614,260	300	2,527
	K75^a^	4,167,451	1,308,301,284	300	2,578
	K80^a^	4,007,361	1,244,363,080	300	4,366
	Trinity	1,017,500	448,075,968	469	16,795

**Notes.**

a The k-mer sizes used in SOAP denovo2.

### Mitogenomic sequences and annotation

**Figure 1:**
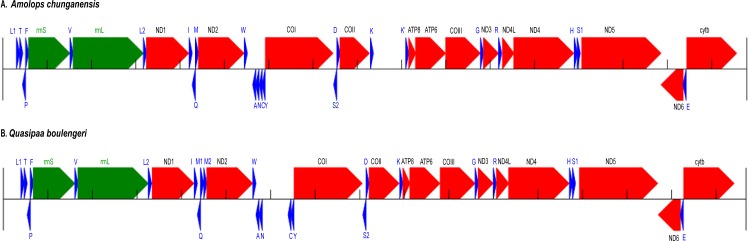
Annotation and map of *Amolops chunganensis* and *Quasipaa boulengeri*. Annotation of *Amolops chunganensis* (A) and *Quasipaa boulengeri* (B) mitogenome. PCGs are colored in red, tRNA-coding genes are in blue, *rrnL* and *rrnS* are in green. A pseudogene of *trnK* is located between *cox2* and *atp8* genes. Each gene is shown as an arrow indicating the transcription direction. The arrows on top of the black line correspond to genes coded on the H-strand, and those below show genes on the L-strand.

We recovered the nearly complete mitogenome of two species, *A. chunganensis* and *Q. boulengeri*. For the mitochondrial genome of *A. chunganensis*, this is the first work reporting the nearly complete mitochondrial genome. The nearly complete mitogenome sequences of *A. chunganensis* and *Q. boulengeri* were 16,795 bp and 16,672 bp in total length, respectively (GenBank accession no. KX645666 and KX645665). The mitogenome of *A. chunganensis* contained 22 transfer RNAs, 2 ribosomal RNAs, 13 PCGs and partial D-loop region (Table S2). Comparatively, the mitogenome of *Q. boulengeri* contained 23 transfer RNAs with a tandem duplication of *trnM* (Table S3). The annotation and gene map of two mitogenomes were illustrated in Fig. 1. In these mitogenomes, eight tRNAs and *nad6* gene were encoded in the L-strand, and the rest of genes were encoded in the H-strand (Fig. 1; Tables S2 and S3), as described in other anurans (Shan et al., 2016; Shan et al., 2014; Zhang et al., 2013). The tRNA-coding genes length ranged from 60 to 73 bp. Secondary structures developed by MITOS suggested that all tRNAs fold in a cloverleaf structure (Figs. S1 and S2).

In *A. chunganensis* mitogenome, ten of 13 PCGs started with the common initiation codon ATG, while *nad2* began with ATT, *cox1* and *atp6* initiated with ATA (Table S2). For the terminal codon, *atp8*, *cox1* and *nad6* genes stop with TAG, AGG and AGA, respectively; *nad4l*, *nad4* and *cytb* ended with TAA; while *nad2* and *atp6* ended with TA. Five PCGs (*cox2*, *cox3* and *nad1, nad3, nad5*) ended with an incomplete stop codon (T–). In addition, we had checked two repeated units 5′-CCGTATGGTTTAATT -ATATACCATCTAATAGGTGATATATATACA-3′ (8 times) and 5′-CCTATATATGCC -CGATATATACTATACTAAGTATTAATCA-3′ (6 times) located at the upstream of *trnL* and the downstream of *cytb*, respectively. Because of the tandem repeat units, only 530 bp had been recovered in the D-loop region for *A. chunganensis*.

In *Q. boulengeri* mitogenome, eleven of 13 PCGs started with ATG, and the *nad2* and *cox1* initiated with ATT and ATA, respectively (Table S3). There were four kinds of stop codon in the 13 PCGs: AGG (*cox1* and *nad6*), TAA (*atp8*, *cytb* and *nad4l*), TAG (*nad5*) and incomplete codon T–in all other seven genes. The incomplete stop codon (T–) is presumably completed as TAA by post transcriptional polyadenylation (Huang et al., 2014; Ojala, Montoya & Attardi, 1981). Overall nucleotide compositions of those mtDNA sequences were A (28.5%), G (14.8%), T (30.1%), and C (26.5%) in *A. chunganensis*, and A (27.8%), G (14.8%), T (28.3%), and C (29.1%) in *Q. boulengeri*, respectively. Moreover, we had checked one repeated unit 5′-TTTTAAGTTA-3′ (25 times) located at the upstream of *trnL*. Similarly, these multiple tandem repeated units caused the incomplete assembly of the D-loop region in *Q. boulengeri*.

### Gene rearrangement

The gene order in *A. chunganensis* and *Q. boulengeri* follows the typical organization of neobatrachians, except some rearrangements of tRNA genes (Zhang et al., 2005; Zhang et al., 2013; Kurabayashi & Sumida, 2013). Compared with reported ranid frogs, the location of replication origin (O_L_) in *A. chunganensis* mitogenome was rearranged. This gene order was consistent with other reported *Amolops* species (Zhang, Xia & Zeng, 2016; Xue et al., 2015; Xia et al., 2014). The O_L_ is located in the tRNA genes cluster “WANCY”. The typical gene order of the “WANCY” cluster is *trnW*, *trnA*, *trnN*, O_L_, *trnC* and *trnY*.

In *A. chunganensis*, however, we found O_L_ was located between the *trnW* and *trnA*. It is similar to *A. mantzorum* and *A. loloensis* gene arrangement pattern (Shan et al., 2016; Xue et al., 2015). Interestingly, a *trnK* pseudogene was located between *cox2* and *atp8* genes (Fig. 1A; Table S2). Compared with the *trnK* gene, the pseudogene has a nucleotide deletion in the anticodon loop, and has some mutations in the acceptor stem of its secondary structure (Table S2 and Fig. S1). Additionally, the W-O_L_-ANCY structure from assembled NGS reads was consistent with previous reported gene rearrangement pattern of another individual of *A. chunganensis* (KF771328; Xia et al., 2014).

In the mitogenome of *Q. boulengeri*, two *trnM* genes were derived from a tandem duplication, which was consistent with other *Quasipaa* species (Chen et al., 2015; Shan et al., 2014; Zhou et al., 2009). Moreover, pseudogene or residues of O_L_ and *trnN* were observed in the tRNA genes cluster “WANCY” (between *trnW* and *trnY* ), resulting to rearranged pattern: “WAN-O_L_-N’-O_L_’-CY” in this individual Furthermore, the gene order of the two available *Q. boulengeri* individuals (GenBank no. KF199152, KC686711; Table S1) were quite different in the “WANCY” region. This indicated that the intraspecific diversity of gene rearrangements existed in *Q. boulengeri* mitogenomes. In addition, 576 non-coding nucleotides were observed between *trnN* and *trnC* (Fig. 1B), which is similar to other individuals of *Q. boulengeri* by Sanger sequencing (Xia et al., 2016). These observations supported that mitogenome with gene order rearrangement can be obtained effectively and correctly from assembled NGS reads.

### Phylogenetic analysis

The concatenated PCGs data of mitogenome sequences contained 11,502 nucleotide positions, including 3,630 conserved sites, 7,806 variable sites and 7,068 potentially parsimony-informative sites. Bayesian inference and Maximum likelihood methods with GTR + I + G model consistently supported the same topology.

**Figure 2:**
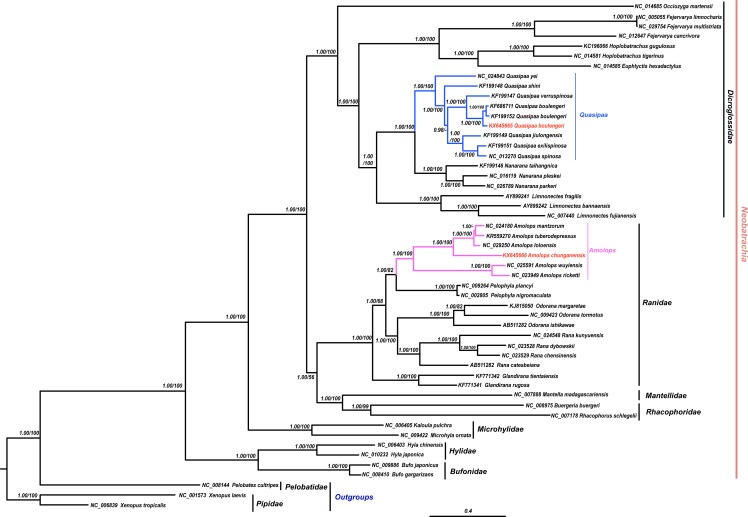
The Bayesian Inference (BI) and Maximum Likelyhood (ML) tree (combined 13 PCGs, 11,502 bp). The Bayesian Inference (BI) and Maximum Likelihood (ML) tree based on nucleotide sequences of the combined 13 protein-coding genes (11,502 in size). For the BI tree, the GTR + I + G model was selected, and two independent runs were performed for 1,000,000 generations with sampling frequency 0.001. The GenBank accession numbers of all species are shown. Numbers beside the nodes are Bayesian posterior probabilities and ML Bootstrap, respectively (showed in BPP/BS). between *cox2* and *atp8* genes. Each gene is shown as an arrow indicating the transcription direction. The arrows on top of the black line correspond to genes coded on the H-strand, and those below show genes on the L-strand.

The results of phylogenetic analysis revealed that monophyly of the Neobatrachia was well supported by our work and previous molecular studies (Xia et al., 2014; Roelants et al., 2007; Frost et al., 2006). The family Dicroglossidae was the sister clade to the clade ((Rhacophoridae + Mantellidae) + Ranidae) (Fig. 2). In phylogenetic tree, all *Amolops* and *Quasipaa* species formed a monophyly, respectively. The *Amolops* clade was split into two sub-clades. One sub-clade contained *A. ricketti* and *A. wuyiensis*, and the other one included *A. chunganensis*, *A. loloensis*, *A. mantzorum* and *A. tuberodepressus* (Fig. 2). The molecular phylogenetic result was consistent with the previous studies (Zhang, Xia & Zeng, 2016; Lv, Bi & Fu, 2014; Cai et al., 2007). Three *Q. boulengeri* individuals formed a monophyletic group, sister to *Q. verrucospinosa*. Clade *Quasipaa* comprised *Q. yei* as the sister taxon to the sub-clade (100%, 1.00) containing (*Q. shini* + ((*Q. jiulongensis* + (*Q. spinosa* + *Q. exilispinosa*)) + (*Q. boulengeri* + *Q. verrucospinosa*))) (Fig. 2). The phylogenetic relationships supported the authenticity of the two obtained mitogenomes among neobatrachians.

## Discussion

### Efficient assembly of mitochondrial genomes

The length of the best fitted contigs for *A. chunganensis* and *Q. boulengeri* was 16,795 bp and 16,672 bp, respectively. We failed to recover the complete mitogenome of *A. chunganensis* and *Q. boulengeri*, due to the highly repetitive sequences in the D-loop region. Except the gap in the D-loop region, the whole mitogenomes were assembled successfully even if there are gene rearrangement in both species. Repeated regions are a well-known problem for sequence assembly algorithms, and it was hard to assemble the D-loop region with extensive repeated regions by NGS (Hahn, Bachmann & Chevreux, 2013; Tang et al., 2014). In the D-loop region, different repeated units, repetitive sequence and repetitions resulted in different sequence length (length polymorphism). For example, in *Amolops* species, the D-loop region ranged from 2,211 bp (*A. mantzorum*) to 3,391 bp (*A. loloensis*) (Zhang, Xia & Zeng, 2016; Shan et al., 2014; Xue et al., 2015). Actually, even if long tandem repeat regions can be successfully amplified by long PCR, they also represent a problem for Sanger sequencing (Xia et al., 2014).

Gene order rearrangement is not a big problem for the assembly of mitogenomes by NGS. In the assembly process, the highly covered regions may be removed if uniform coverage of reads are assumed by a *de novo* genome assembler (Rubinstein et al., 2013). This may be disadvantageous to the assembly of mitogenomes with gene order rearrangement. Tandem duplication followed by random loss (TDRL) is the most frequently invoked model to explain the diversity of gene rearrangements in metazoan mitogenomes (Boore, 2000). According to this model, two copies of mitochondrial gene fragment would exist after tandem duplication. These tandem duplications would cause errors when using short reads of NGS to assemble mitogenomes. Nevertheless, deletion of a redundant gene-copy may happen rapidly due to replication and/or transcription efficiency, facilitating the formation of pseudogenes or the complete deletion of redundant genes Such pseudogenes or residues of tandem repeats could be efficiently distinguished from the original copy.

By aligning published mitogenomes of close species and individuals in rRNAs, tRNAs and PCGs, we assessed the quality of our assembled data. The alignment results suggested that the assembled sequences were consistent with majority of other anurans mitogenomes The non-conservative regions were located between *nad2* and *cox1*, which includes gene rearrangements in *Amolops* and *Quasipaa* reported previously (Xia et al., 2014; Shan et al., 2014; Shan et al., 2016).

Our results illustrated that mitochondrial sequences can be successfully assembled from the NGS raw data. Compared with traditional methods, the strategy of mitogenomes assembly from NGS raw data has two distinct advantages. First, it neither depends on specific primers nor be affected by gene rearrangement. When gene rearrangement region include primers, the PCR-based method could not be successfully amplified. Second, this approach is fast, timesaving, relatively cheap, and does not require great effort to recover the mitochondrial genomes (Gan, Schultz & Austin, 2014; Tang et al., 2014; Rubinstein et al., 2013; Haiminen et al., 2011). However, the disadvantage of such an approach is that it requires constructing separate genomic libraries for each sample.

### Mitochondrial gene order rearrangements

Some pseudogenes or residues of O_L_ *trnK* and *trnN* were observed in *A. chunganensis* and *Q. boulengeri*. These gene rearrangements may be explained by the theory of TDRL (Mueller & Boore, 2005; Boore, 2000). In *A. chunganensis*, O_L_ was located between *trnW* and *trnA*. For *Q boulengeri*, the intraspecific gene rearrangements were checked in the WANCY region. Our results supported the observation that the hotspot of gene rearrangement was adjacent to the origin of light-strand replication (San Mauro et al., 2006). The widespread occurrence of gene rearrangement among different vertebrate groups has been examined in relation to variability in the thermodynamic stability of the light-strand replication origin (Fonseca & Harris, 2008).

Mitochondrial metagenomic skimming by NGS could be a useful approach to recover intraspecific rearrangements of mitogenomes. By sequencing the hotspot of gene rearrangement of mtDNA for *Q boulengeri* (from *nad2* to *cox1*, which includes the WANCY region), four kinds of gene rearrangements were identified in spiny-bellied frog populations (Xia et al., 2016). Yet, mitochondrial genome analysis may provide more valuable evidences for interpreting the intraspecific gene rearrangements. Intraspecific rearranged variations were not common by comparison of all mtDNA records of amphibians and reptiles in GenBank.

### Phylogenetic reconstruction of mitogenomic data

In this study, both the phylogenetic relationships and mitogenome organization suggest that *A. wuyiensis* and *A. ricketti* were significantly distinguished from other *Amolops* species (Fei et al., 2009). Within *Amolops* clade, the highly supported sister relationship of *A. wuyiensis* and *A. richetti* was consistent with the results of Cai et al. (2007). Within *Quasipaa* clade, the phylogenetic inferences based on mtDNA sequences revealed that all individuals of *Q. boulengeri* formed a monophyly with high supports, sister to *Q. verruspinosa*. This result is congruent with Che et al. (2009), but different from the result of Qing et al. (2012). The phylogenetic reconstruction using whole mitogenome provides more credible result than single gene.

As previous demonstration (Yan et al., 2013; Qing et al., 2012; Cai et al., 2007; Frost et al., 2006), the mitogenomic approach proved useful tools for inferring phylogenetic relationships within Neobatrachia. In the present study, all clades were well resolved, with a few exceptions where Bayesian posterior probabilities were no less than 0.90. Despite their fast evolutionary rate, mitochondrial genomes contained a phylogenetic signal that could be efficiently recovered with reasonable taxon sampling (Rubinstein et al., 2013).

##  Supplemental Information

10.7717/peerj.2786/supp-1 Figure S1 Secondary structure of 22 tRNAs for *Amolops chunganensis*

10.7717/peerj.2786/supp-2 Figure S2 Secondary structure of 23 tRNAs for *Quasipaa boulengeri*

10.7717/peerj.2786/supp-3 Table S1 The information of the samples used in this study

10.7717/peerj.2786/supp-4 Table S2 Location of features in *Amolops chunganensis* mitogenome

10.7717/peerj.2786/supp-5 Table S3 Location of features in *Quasipaa boulengeri* mitogenome

10.7717/peerj.2786/supp-6 Supplemental Information 1 Combined 13 PCGs of 51 sequences

## Additional Information and Declarations

### Field Study Permissions

The following information was supplied relating to field study approvals (i.e., approving body and any reference numbers): All field work with animals was conducted according to relevant national and international guide-lines. Chengdu Institute of Biology issued permit number CIB#2014-36 and CIB#2014-110 for field work.

### DNA Deposition

The following information was supplied regarding the deposition of DNA sequences: The sequences of *Quasipaa boulengeri* and *Amolops chunganensis* are accessible via GenBank accession numbers KX645665 –KX645666 in this study, respectively.

### Data Availability

The following information was supplied regarding data availability: Sequence Read Archive (SRA, http://www.ncbi.nlm.nih.gov/Traces/sra)
SRR3929655 for *Quasipaa boulengeri*;
SRR3929656 for *Amolops chunganensis*.
