# Supplementary figures and images for: Next-generation sequencing of mixed genomic DNA allows efficient assembly of rearranged mitochondrial genomes in Amolops chunganensis and Quasipaa boulengeri
Source: PeerJ. 2016 Dec 15;4:e2786. doi: 10.7717/peerj.2786 (PMC5162401; doi:10.7717/peerj.2786)

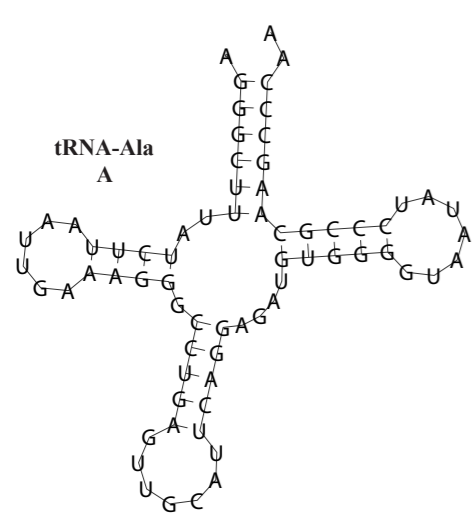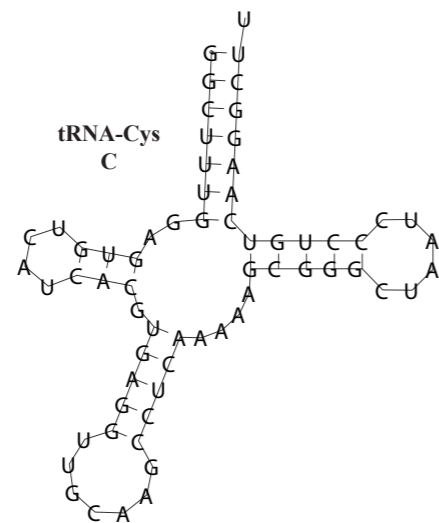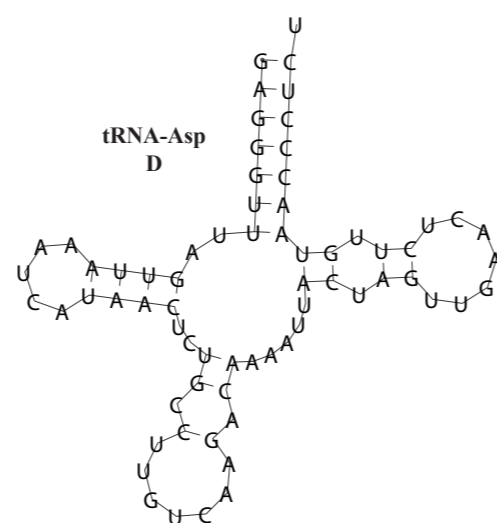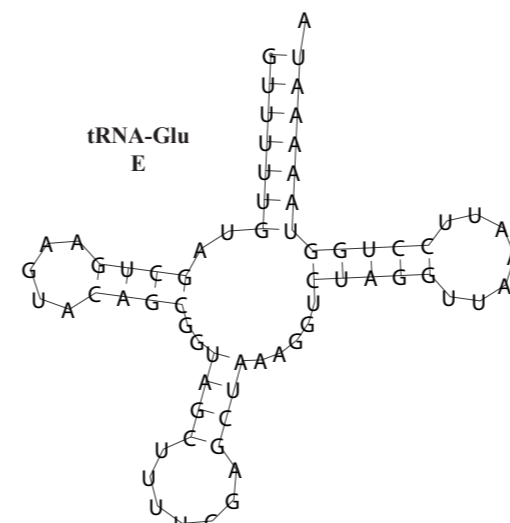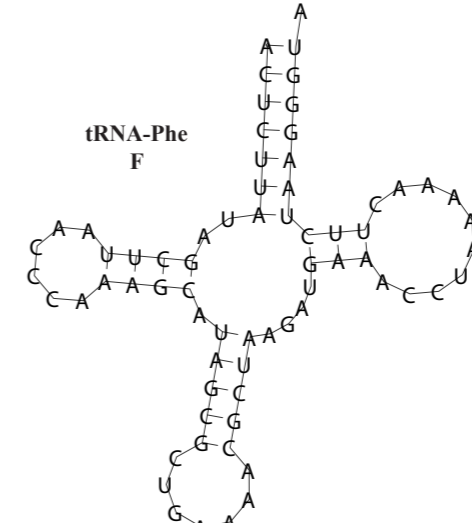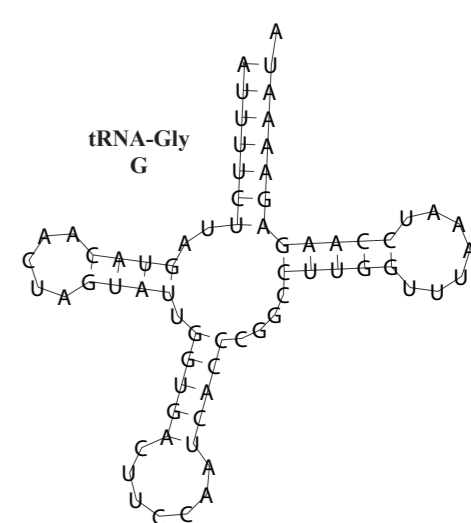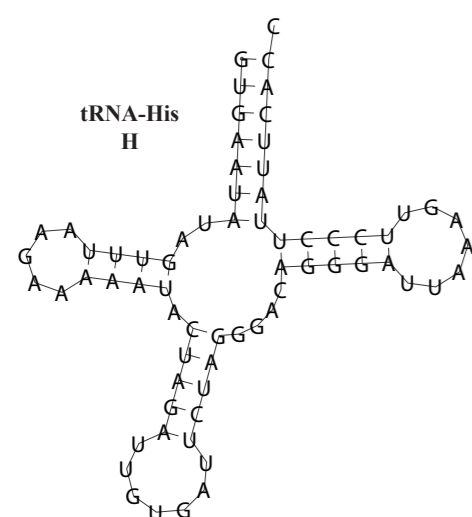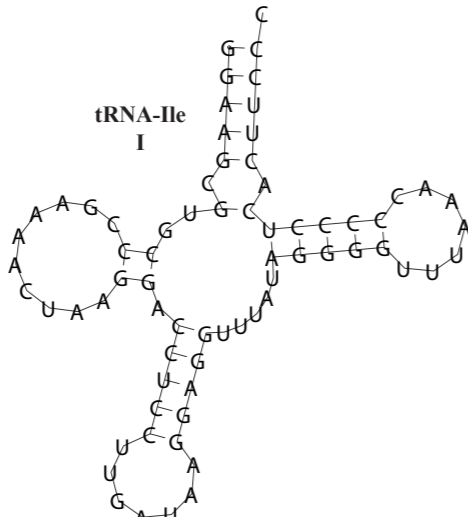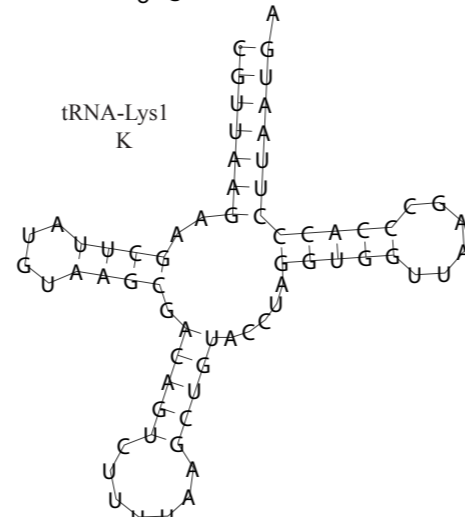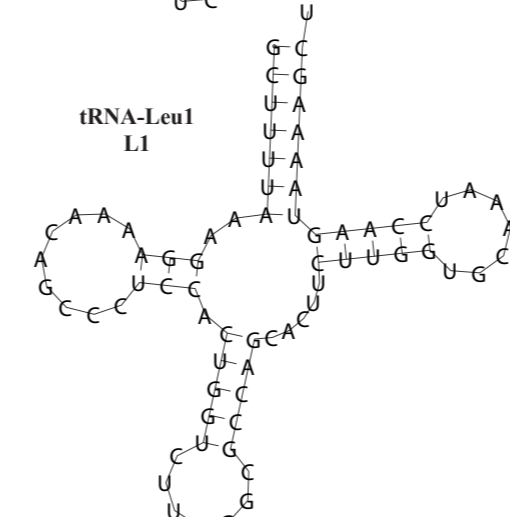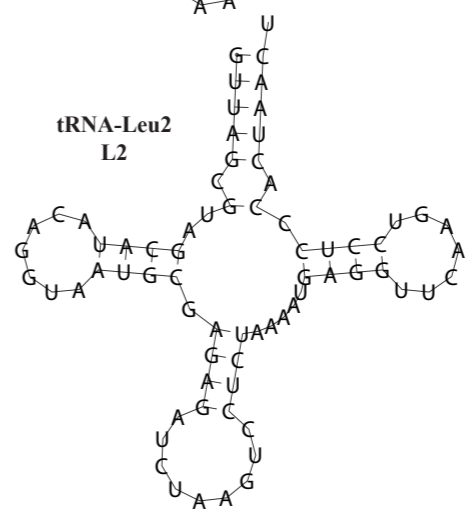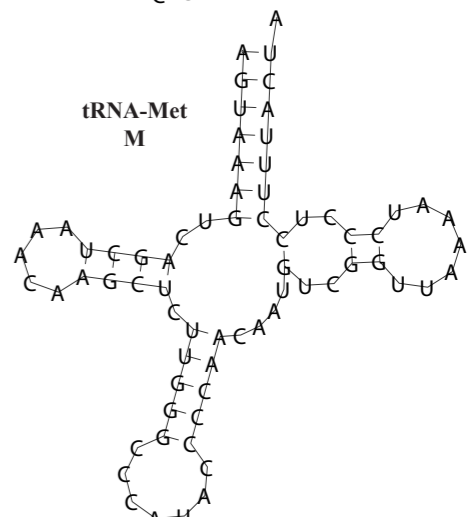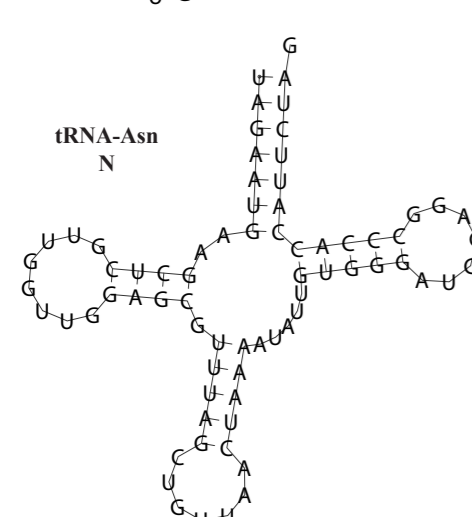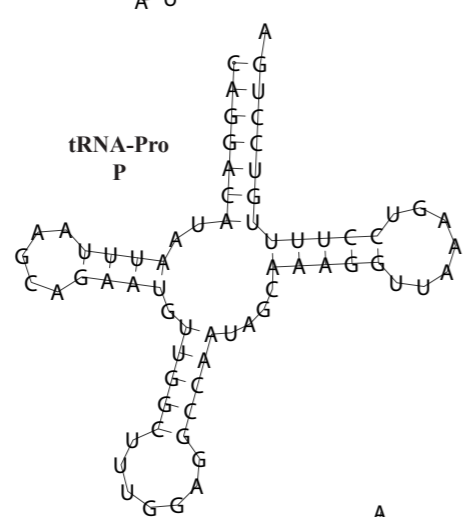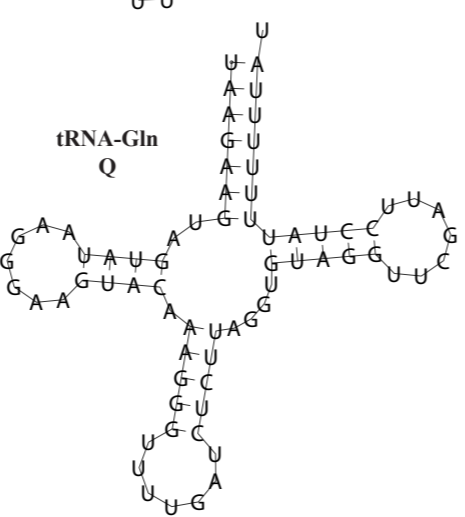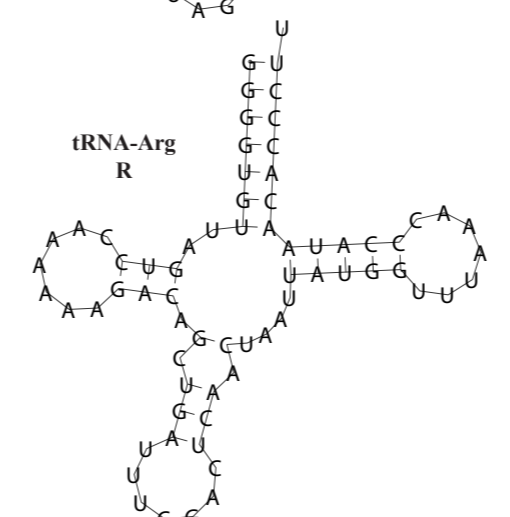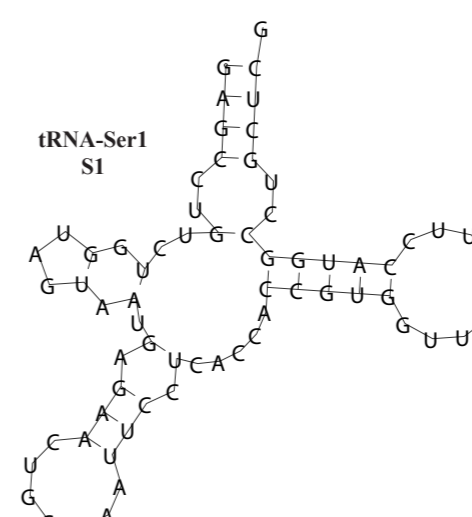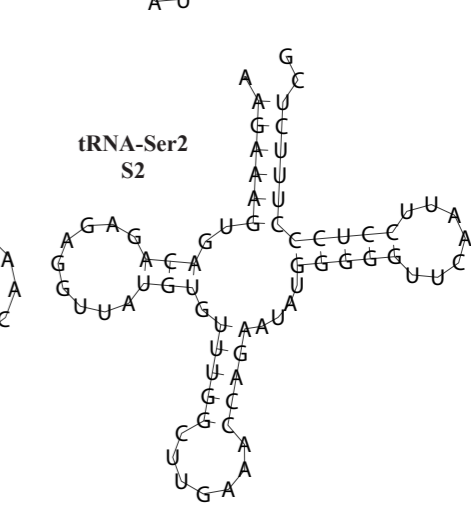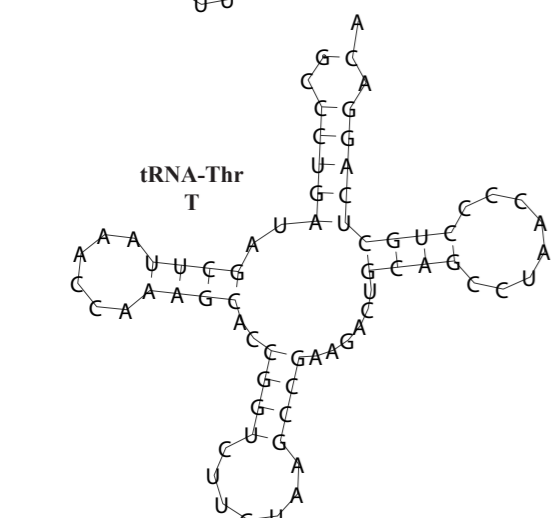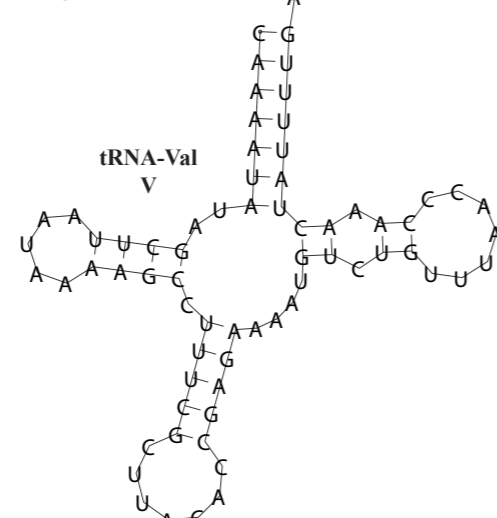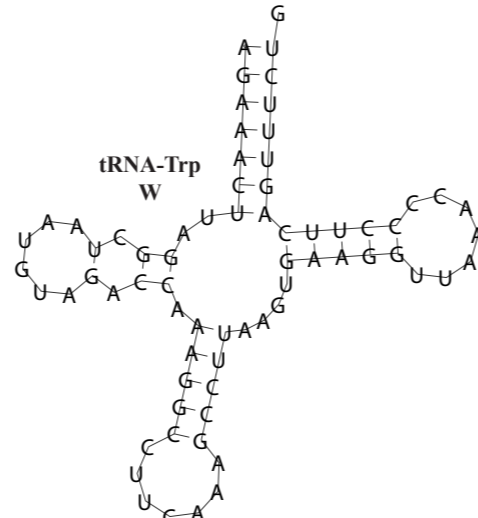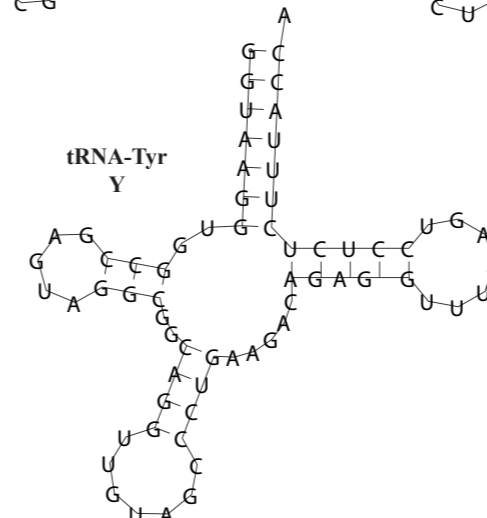

Supplement: Figure S1 [file peerj-04-2786-s001.pdf]

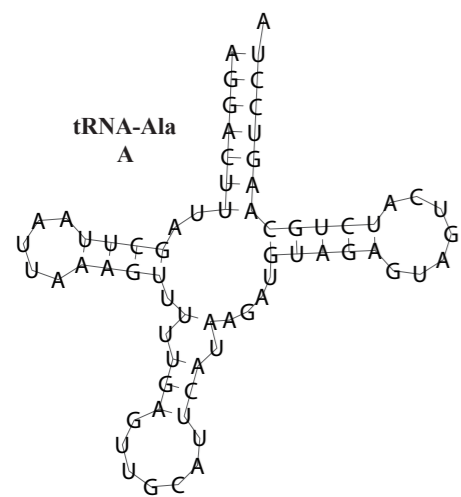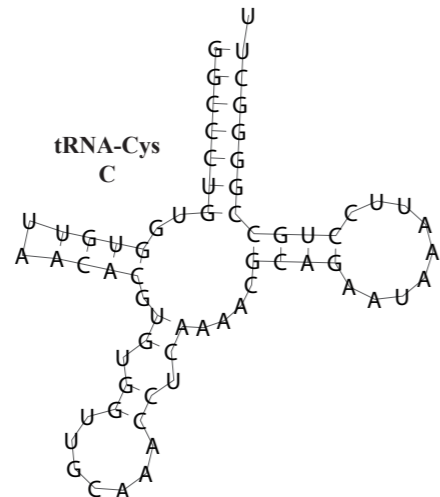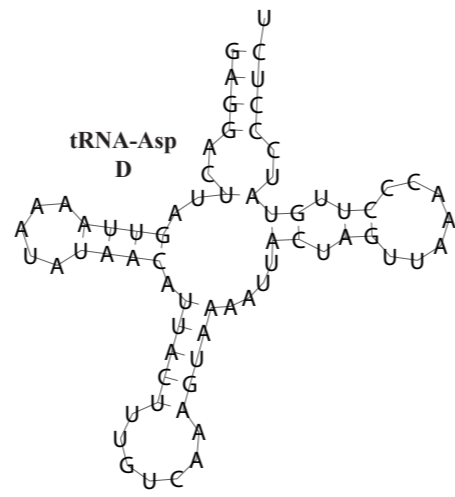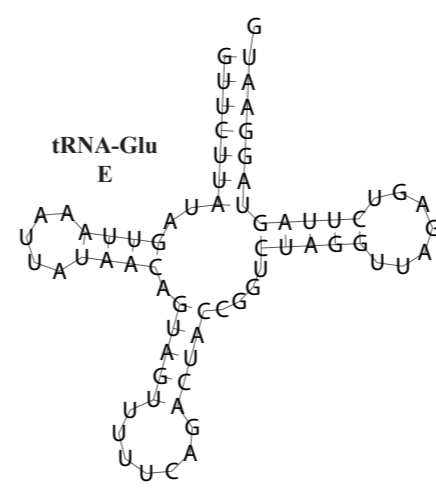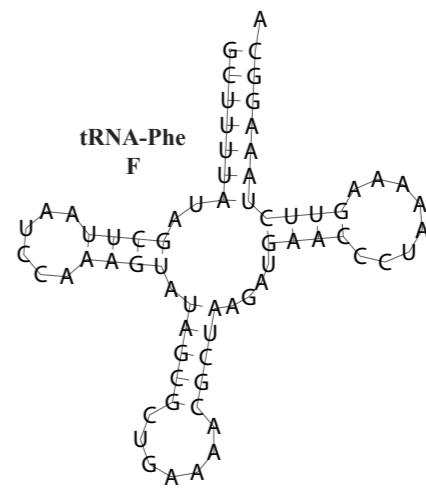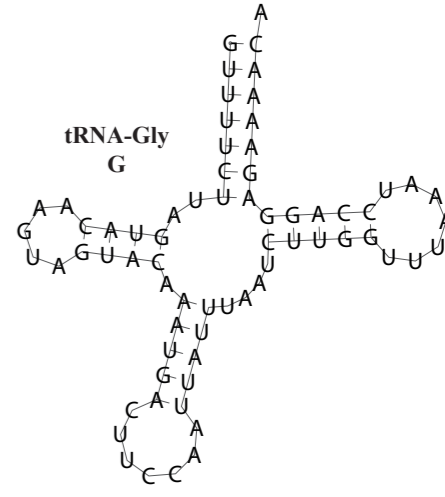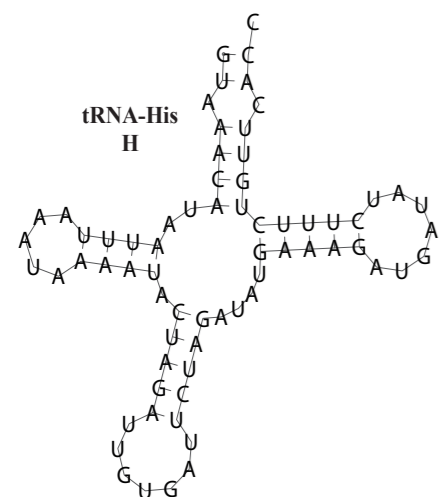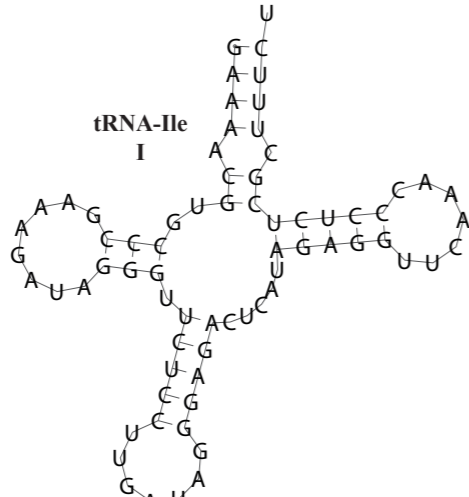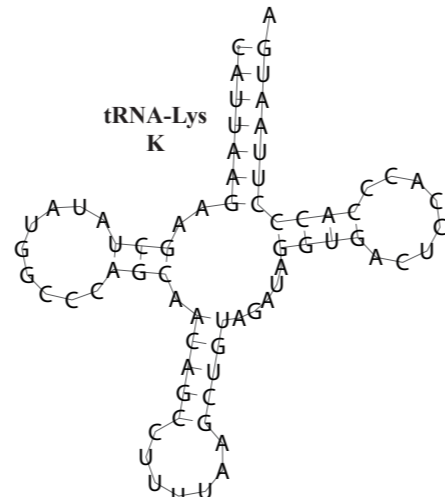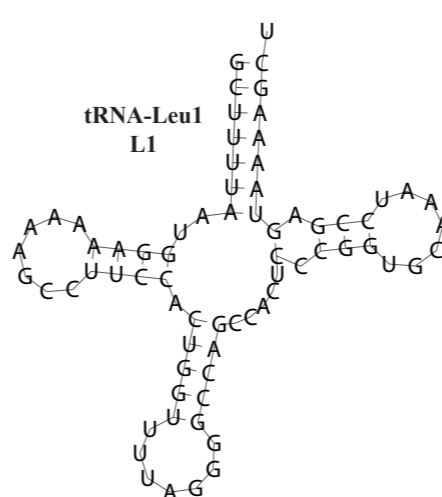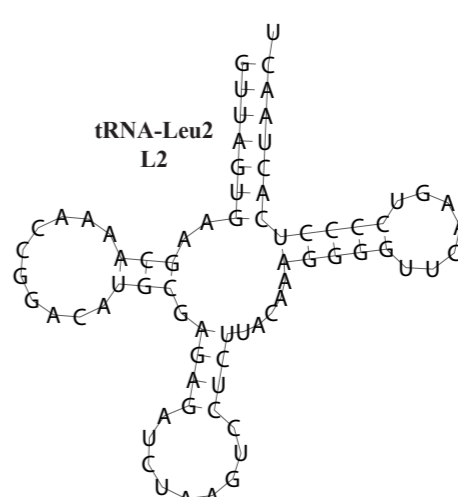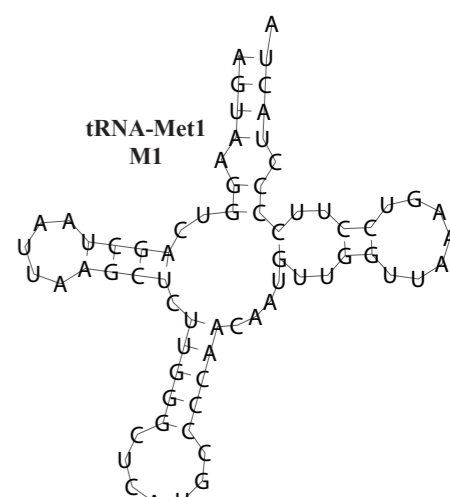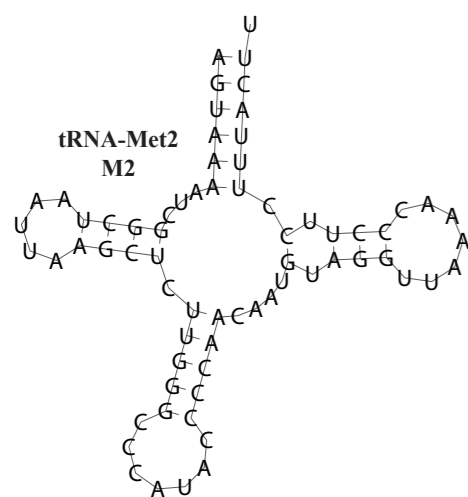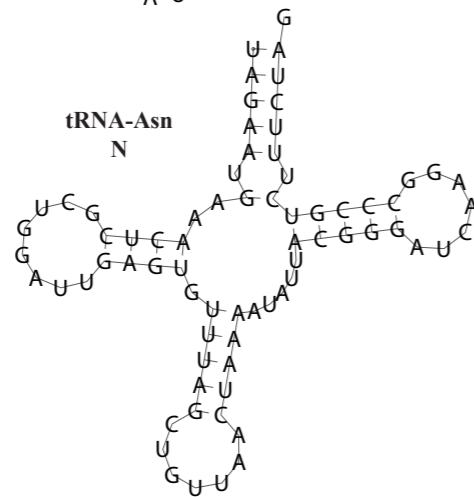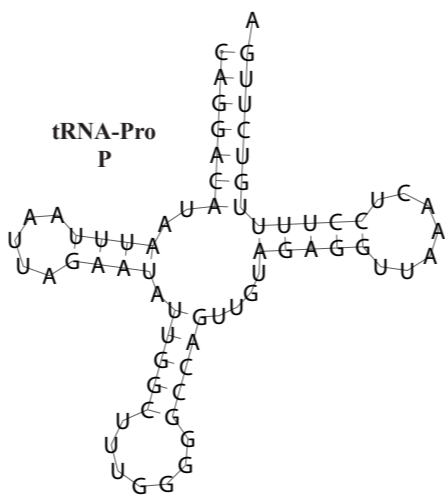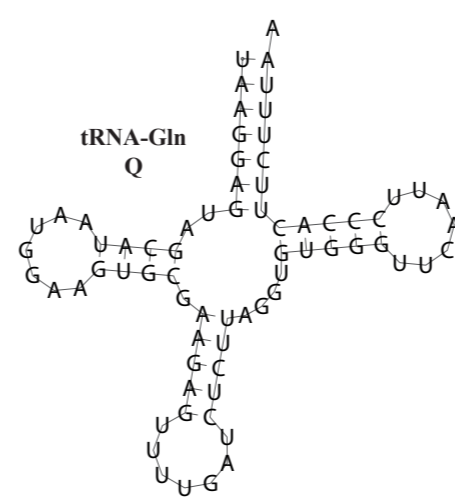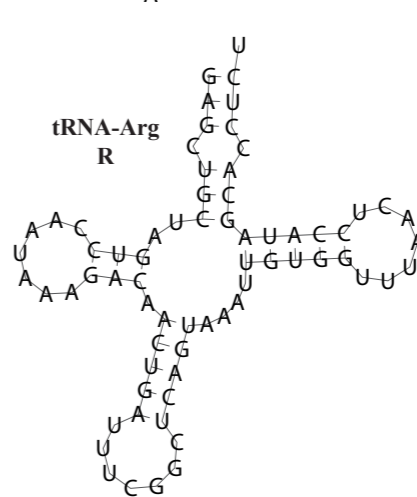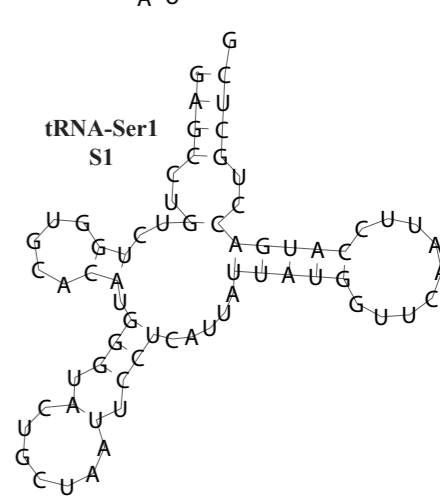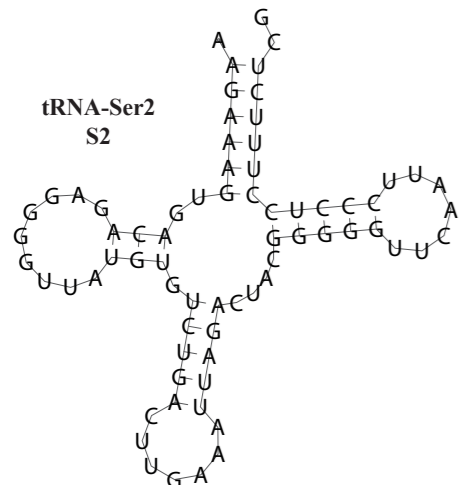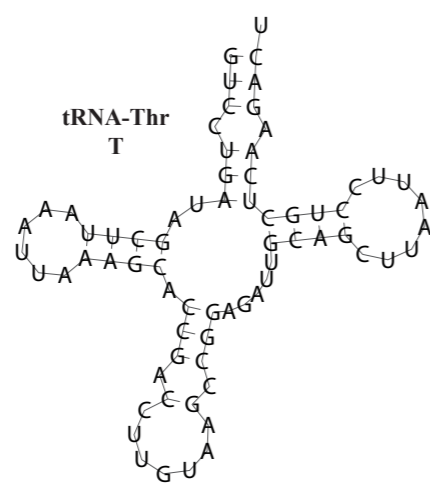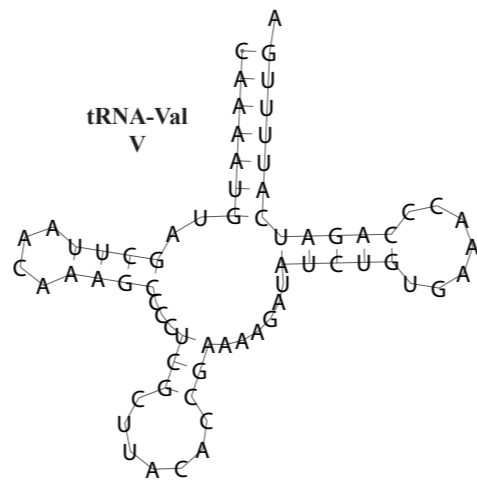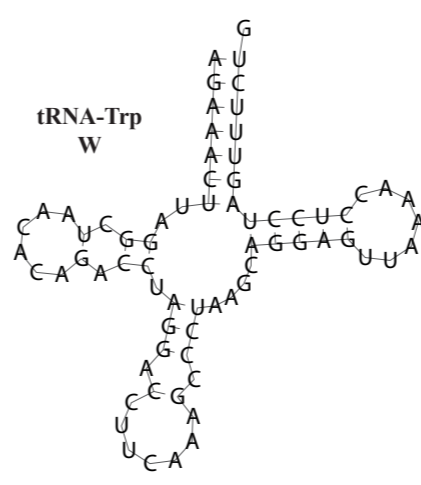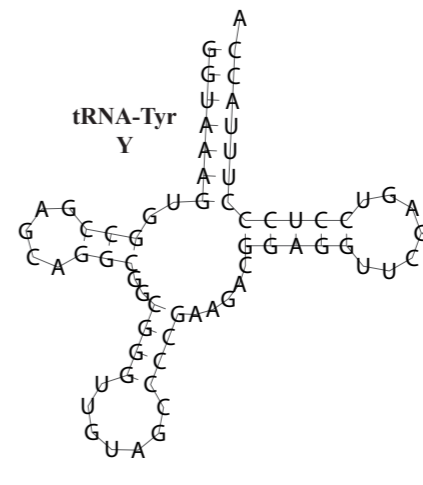

Supplement: Figure S2 [file peerj-04-2786-s002.pdf]
