# Supplementary material for: Next-generation sequencing of mixed genomic DNA allows efficient assembly of rearranged mitochondrial genomes in Amolops chunganensis and Quasipaa boulengeri
Source: PeerJ. 2016 Dec 15;4:e2786. doi: 10.7717/peerj.2786 (PMC5162401; doi:10.7717/peerj.2786)
Supplement: Table S1 [file peerj-04-2786-s003.docx]

Table S1. The information of the samples used in this study.

| Species | Length/bp | GeneBank Accession No. | References |
| --- | --- | --- | --- |
| *Amolops chunganensis* | 16,795 | KX645666 | This study |
| *Amolops loloensis* | 18,926 | NC_029250 | Xue et al., 2015 |
| *Amolops mantzorum* | 17,744 | NC_024180 | Shan et al., 2016 |
| *Amolops ricketti* | 17,772 | NC_023949 | Li et al., 2014a |
| *Amolops tuberodepressus* | 18,348 | KR559270 | Zhang et al., 2015 |
| *Amolops wuyiensis* | 17,797 | NC_025591 | Huang et al., 2014 |
| *Quasipaa boulengeri* | 16,672 | KX645665 | This study |
| *Quasipaa boulengeri* | 15,106 | KF199152 | Zhang and Yu, 2013 (Direct submission) |
| *Quasipaa boulengeri* | 17,741 | NC_021937 | Shan et al., 2014 |
| *Quasipaa exilispinosa* | 14,903 | KF199151 | Zhang et al., 2013 (Direct submission) |
| *Quasipaa jiulongensis* | 15,072 | KF199149 | Zhang and Yu, 2013 (Direct submission) |
| *Quasipaa spinosa* | 18,012 | NC_013270 | Zhou et al., 2009 |
| *Quasipaa shini* | 14,943 | KF199148 | Zhang et al., 2013 (Direct submission) |
| *Quasipaa verruspinosa* | 15,063 | KF199147 | Zhang and Yu, 2013 (Direct submission) |
| *Quasipaa yei* | 17,072 | NC_024843 | Chen et al., 2015 |
| *Odorana tormotus* | 17,962 | NC_009423 | Su et al., 2007 |
| *Odorana margaretae* | 17,903 | KJ815050 | Chen et al., 2014 |
| *Odorrana ishikawae* | 21,020 | AB511282 | Kurabayashi et al., 2010 |
| *Rana catesbeiana* | 17,682 | AB761267 | Kakehashi et al., 2012 |
| *Rana chensinensis* | 18,808 | NC_023529 | Li et al.,2014b |
| *Rana kunyuensis* | 22,255 | NC_024548 | Li et al., 2014c |
| *Rana dybowskii* | 18,864 | NC_023528 | Li et al., 2014b |
| *Hoplobatrachus rugulosus* | 16,903 | KC196066 | Pan et al., 2012 (Direct submission) |
| *Hoplobatrachus tigerinus* | 20,462 | NC_014581 | Alam et al., 2010 |
| *Fejervarya multistriata* | 17,750 | NC_029754 | Huang and Tu, 2016 (Direct submission) |
| *Fejervarya limnocharis* | 17,717 | NC_005055 | Liu, 2005 |
| *Fejervarya cancrivora* | 17,843 | NC_012647 | Ren, 2009 |
| *Nanorana pleskei* | 17,660 | NC_016119 | Chen et al., 2011 |
| *Nanorana parkeri* | 17,837 | NC_026789 | Jiang et al, 2015 |
| *Nanorana taihangnica* | 21,322 | KF199146 | Zhang et al., 2013 (Direct submission) |
| *Limnonectes fujianensis* | 17,654 | NC_007440 | Nie, Hu and Zhang, 2005 (Direct submission) |
| *Limnonectes bannaensis* | 16,867 | AY899242 | Zhang et al., 2009 |
| *Limnonectes fragilis* | 16,640 | AY899241 | Nie and Zhang, 2005 (Direct submission) |
| *Euphlyctis hexadactylus* | 20,280 | NC_014584 | Alam et al., 2010 |
| *Occidozyga martensii* | 18,321 | NC_014685 | Li et al., 2014d |
| *Bufo gargarizans* | 17,277 | NC_008410 | Cao et al., 2006 |
| *Bufo japonicus* | 17,757 | NC_009886 | Igawa et al, 2008 |
| *Hyla chinensis* | 18,180 | NC_006403 | Zhang P., 2005 |
| *Hyla japonica* | 19,519 | NC_010232 | Igawa et al., 2008 |
| *Kaloula pulchra* | 16,818 | NC_006405 | Zhang P., 2005 |
| *Microhyla ornata* | 16,730 | NC_009422 | Nie and Cao, 2007 (Direct submission) |
| *Pelophylax nigromaculata* | 17,804 | NC_002805 | Sumida et al., 2001 |
| *Pelophylax plancyi* | 17,822 | NC_009264 | Nie et al., 2007 (Direct submission) |
| *Glandirana rugosa* | 17,426 | KF771341 | Xia et al., 2014 |
| *Glandirana tientaiensis* | 17,347 | KF771342 | Xia et al., 2014 |
| *Buergeria buergeri* | 19,959 | NC_008975 | Sano, 2004 |
| *Rhacophorus schlegelii* | 21,359 | NC_007178 | Sano, 2005 |
| *Mantella madagascariensis* | 22,874 | NC_007888 | Kurabayashi, 2006 |
| *Xenopus laevis* | 17,553 | NC_001573 | Roe et al., 1985 |
| *Xenopus tropicalis* | 17,610 | NC_006839 | Macey et al., 2004 (Direct submission) |
| *Pelobates cultripes* | 17,384 | NC_008144 | Gissi et al., 2006 |

References

Alam MS, Kurabayashi A, Hayashi Y, Sano N, Khan MR, Fujii T, Sumida M. 2010. Complete mitochondrial genomes and novel gene rearrangements in two dicroglossid frogs, *Hoplobatrachus tigerinus* and *Euphlyctis hexadactylus*, from Bangladesh. Genes Genetics Systems 85 (3), 219-232 DOI http://doi.org/10.1266/ggs.85.219.

Cao SY, Wu XB, Yan P, Hu YL, Su X, Jiang ZG. 2006. Complete nucleotide sequences and gene organization of mitochondrial genome of *Bufo* *gargarizans*. Mitochondrion 6(4):186–193 DOI 10.1016/j.mito.2006.07.003.

Chen GY, Wang B, Liu JY, Xie F, Jiang JP. 2011. Complete mitochondrial genome of *Nanorana pleskei* and evolutional characteristics. Current Zoolgoy, 57(6): 785-805 DOI: DOI: 10.1093/czoolo/57.6.785.

Chen Z, Zhai XF, Zhu YJ, Chen XH. 2015. Complete mitochondrial genome of the Ye’s spiny–vented frog *Yerana yei* (Anura: Dicroglossidae). Mitochondrial DNA 26(3): 489–490 DOI 10.3109/19401736.2014.926542.

Chen Z, Zhang J, Zhai X, Zhu Y, Chen X. 2014a. Complete mitochondrial genome of the green odorous frog *Odorrana margaretae* (Anura: Ranidae). Mitochondrial DNA [Epub ahead of print]. DOI 10.3109/19401736.2014.926533.

Gissi C, San Mauro D, Pesole G, Zardoya R. 2006. Mitochondrial phylogeny of Anura (Amphibia): a case study of congruent phylogenetic reconstruction using amino acid and nucleotide characters. Gene, 366(2):228-237 DOI 10.1016/j.gene.2005.07.034.

Huang M, Duan R, Kong X, Wang H, Zhu H. 2014. The complete mitochondrial genome of *Amolops wuyiensis* (Anura: Ranidae). Mitochondrial DNA [Epub ahead of print]. DOI 10.3109/ 19401736.2014.961131.

Huang Z, Tu F. 2016. The complete mitochondrial genome of *Fejervarya multistriata*. Direct submission。

Igawa T, Kurabayashi A, Usuki C, Fujii T, Sumida M. 2008. Complete mitochondrial genomes of three neobatrachian anurans: a case study of divergence time estimation using different data and calibration settings. Gene, 407(1-2):116-29 DOI 10.1016/j.gene.2007.10.001.

Jiang LC, Ruan QP, Chen W. 2015. The complete mitochondrial genome sequence of the Xizang Plateau frog, *Nanorana parkeri* (Anura: Dicroglossidae). Mitochondrial DNA, 1-2 DOI 10.3109/19401736.2015.1007327.

Kakehashi R, Kurabayashi A, Oumi S, Katsuren S, Hoso M, Sumida M. 2013. Mitochondrial genomes of Japanese *Babina* frogs (Ranidae, Anura): unique gene arrangements and the phylogenetic position of genus *Babina*. Genes & Genetic Systems, 88(1): 39-51 DOI http://doi.org/10.1266/ggs.88.59.

Kurabayashi A, Usuki C, Mikami N, Fujii T, Yonekawa H, Sumida M, Hasegawa M. 2006. Complete nucleotide sequence of the mitochondrial genome of a Malagasy poison frog *Mantella madagascariensis*: evolutionary implications on mitochondrial genomes of higher anuran groups. Molecular Phylogenetics and Evolution 39(1): 223–236 DOI 10.1016/j.ympev.2005.11.021.

Kurabayashi A, Yoshikawa N, Sato N, Hayashi Y, Oumi S, Fujii T, Sumida M. 2010. Complete mitochondrial DNA sequence of the endangered frog *Odorrana ishikawae* (family Ranidae) and unexpected diversity of mt gene arrangements in ranids. Molecular Phylogenetics and Evolution 56 (2): 543-553 DOI 10.1016/j.ympev.2010.01.022.

Li E, Li XQ, Wu XB, Feng G, Zhang M, Shi HT, Wang LJ, Jiang JP. 2014d. Complete nucleotide sequence and gene rearrangement of the mitochondrial genome of *Occidozyga martensii*. Journal of Genetics, 93(3): 631-641 DOI 10.1007/s12041-014-0418-4.

Li J, Lei G, Fu C.2014b.Complete mitochondrial genomes of two brown frogs, *Rana dybowskii* and *Rana cf. chensinensis* (Anura: Ranidae).Mitochondrial DNA [Epub ahead of print]. DOI 10.3109/19401736.2013.878921.

Li J., Yin W., Xia R., Lei G., Fu C.2014c. Complete mitochondrial genome of a brown frog, *Rana kunyuensis* (Anura: Ranidae). Mitochondrial DNA [Epub ahead of print]. DOI 10.3109/19401736.2013.869681.

Li Y, Wu X, Zhang H, Yan P, Xue H, Wu X.2014a.The complete mitochondrial genome of *Amolops ricketti* (Amphidia, Anura, Ranidae). Mitochondrial DNA [Epub ahead of print]. DOI 10.3109/19401736.2014.883606.

Liu ZQ, Wang YQ, Su B. 2005. The mitochondrial genome organization of the rice frog, *Fejervarya limnocharis* (Amphibia: Anura): a new gene order in the vertebrate mtDNA. Gene 346, 145–151 DOI 0.1016/j.gene.2004.10.013.

Nie LW, Cao CH, Song JL. 2007. The complete mitochondrial genome of *Rana plancyi* (Amphibia:Anura) and implication for higher Anuran groups phylogeny. Direct submission.

Pan T, Ding L, Huang J, Hang D, Liu Z, Zhang B. 2012. Mitochondrial genome of the *Hoplobatrachus rugulosus*. Direct submission.

Ren Z, Zhu B, Ma E, Wen J, Tu T, Cao Y, Hasegawa M, Zhong Y. 2009. Complete nucleotide sequence and gene arrangement of the mitochondrial genome of the crab-eating frog *Fejervarya cancrivora* and evolutionary implications. Gene 441:148–155 DOI 10.1016/j.gene.2008.09.010.

Roe BA, Ma DP, Wilson RK, Wong JF. 1985. The complete nucleotide sequence of the *Xenopus laevis* mitochondrial genome. The Journal of Biological Chemistry, 260(17):9759-9574.

Sano N, Kurabayashi A, Fujii T, Yonekawa H, Sumida M, 2004. Complete nucleotide sequence and gene rearrangement of the mitochondrial genome of the bell-ring frog, *Buergeria buergeri* (family Rhacophoridae). Genes Genetic Systems 79(3): 151–163 DOI http://doi.org/10.1266/ggs.79.151.

Sano N, Kurabayashi A, Fujii T, Yonekawa H, Sumida M, 2005. Complete nucleotide sequence of the mitochondrial genome of Schlegel's tree frog *Rhacophorus schlegelii* (family Rhacophoridae): duplicated control regions and gene rearrangements. Genes Genetic Systems 80(3), 213–224 DOI http://doi.org/10.1266/ggs.80.213.

Shan X, Xia Y, Kakehashi R, Kurabayashi A, Zou FD, Zeng XM. 2016. Complete mitochondrial genome of *Amolops mantzorum* (Anura: Ranidae). Mitochondrial DNA Part A 27(1): 705–707 DOI 10.3109/ 19401736.2014.913152.

Shan X, Xia Y, ZhengYC, Zou FD, Zeng XM. 2014. The complete mitochondrial genome of *Quasipaa boulengeri* (Anura: Dicroglossidae). Mitochondrial DNA 25(2): 83–84 DOI 10.3109/19401736.2013.782023

Su X, Wu XB, Yan P, Cao SY, Hu YL.2007.Rearrangement of a mitochondrial tRNA gene of the concave-eared torrent frog, *Amolops tormotus*.Gene 394 (1-2): 25-34 DOI 10.1016/j.gene.2007.01.022.

Sumida M, Kanamori Y, Kaneda H, Kato Y, Nishioka M, Hasegawa M, Yonekawa H. 2001. Complete nucleotide sequence and gene rearrangement of the mitochondrial genome of the Japanese pond frog *Rana nigromaculata*. Genes & Genetic Systems, 76(5):311-325 DOI http://doi.org/10.1266/ggs.76.311.

Xia Y, Zheng Y, Miura I, Wong PB, Murphy RW, Zeng X. 2014. The evolution of mitochondrial genomes in modern frogs (Neobatrachia): nonadaptive evolution of mitochondrial genome reorganization. BMC Genomics 15:691 DOI 10.1186/1471-2164-15-691.

Xue R, Liu JB, Yu JJ, Yang JD. 2015. The complete mitogenome of *Amolops loloensis* and related phylogenetic relationship among Ranidae. Mitochondrial DNA Early Online: 1–2 DOI 10.3109/19401736.2015.1101589

Zhang CH, Xia Y, Zeng XM. 2016. Characterization of the mitochondrial genome of *Amolops tuberodepressus* (Anura: Ranidae). Mitochondrial DNA Part A 27(4): 2893-2894 DOI 10.3109/19401736.2015.1060425.

Zhang JF, Nie LW, Wang Y, Hu LL. 2009. The complete mitochondrial genome of the large-headed frog, *Limnonectes bannaensis* (Amphibia: Anura), and a novel gene organization in the vertebrate mtDNA. Gene, 442(1-2): 119-127 DOI 10.1016/j.gene.2009.04.018.

Zhang JY, Yu DN, Lin YB，Zheng RQ. 2013. Complete mitochondrial DNA sequence of *Nanorana taihangnica* (Family Dicroglossidae) and unexpected diversity of mt gene arrangements in tribe *Paini*. Direct submission.

Zhang JY，Yu DN. 2013. Complete mitochondrial DNA sequence of *Nanorana taihangnica* (Family Dicroglossidae) and unexpected diversity of mt gene arrangements in tribe *Paini*. Direct submission.

Zhang P, Zhou H, Chen YQ, Liu YF, Qu LH. 2005. Mitogenomic perspectives on the origin and phylogeny of living amphibians. Systematic Biology 54(3): 391– 400 DOI 10.1080/10635150590945278.

Zhou Y, Zhang JY, Zheng RQ, Yu BG, Yang G. 2009. Complete nucleotide sequence and gene organization of the mitochondrial genome of *Paa spinosa* (Anura: Ranoidae). Gene 447 (2): 86–96 DOI 10.1016/j.gene.2009.07.009.
