# Supplementary material for: Next-generation sequencing of mixed genomic DNA allows efficient assembly of rearranged mitochondrial genomes in Amolops chunganensis and Quasipaa boulengeri
Source: PeerJ. 2016 Dec 15;4:e2786. doi: 10.7717/peerj.2786 (PMC5162401; doi:10.7717/peerj.2786)
Supplement: Table S2 [file peerj-04-2786-s004.docx]

Table S2. Location of features in the mtDNA of *Amolops chunganensis*.

| Name | Amino acids | Position | | Stranad | Length | codon | | Intergenic nucleotides # |
| --- | --- | --- | --- | --- | --- | --- | --- | --- |
|  |  | From | To |  |  | Start | Stop |  |
| D-loop |  | 1 | 309 |  | 309 |  |  |  |
| trnL1 (tag) | Leu | 310 | 381 | H | 72 |  |  |  |
| trnT (tgt) | Thr | 384 | 453 | H | 70 |  |  | +2 |
| trnP (tgg) | Pro | 446 | 514 | L | 69 |  |  | –6 |
| trnF (gaa) | Phe | 516 | 584 | H | 69 |  |  | +1 |
| rrnS |  | 585 | 1518 | H | 934 |  |  |  |
| trnV (tac) | Val | 1520 | 1588 | H | 69 |  |  | +1 |
| rrnL |  | 1589 | 3174 | H | 1586 |  |  |  |
| trnL2 (taa) | Leu | 3175 | 3245 | H | 71 |  |  |  |
| nad1 |  | 3246 | 4206 | H | 960 | ATG | T* |  |
| trnI (gat) | Ile | 4207 | 4277 | H | 71 |  |  |  |
| trnQ (ttg) | Gln | 4278 | 4348 | L | 71 |  |  |  |
| trnM (cat) | Met | 4348 | 4416 | H | 69 |  |  | –1 |
| nad2 |  | 4417 | 5452 | H | 1035 | ATT | TA* |  |
| trnW (tca) | Trp | 5453 | 5522 | H | 70 |  |  |  |
| rep_origin |  | 5600 | 5629 | L | 30 |  |  | +77 |
| trnA (tgc) | Ala | 5643 | 5712 | L | 70 |  |  | +13 |
| trnN (gtt) | Aln | 5713 | 5785 | L | 73 |  |  |  |
| trnC (gca) | Cys | 5793 | 5857 | L | 65 |  |  | +7 |
| trnY (gta) | Tyr | 5858 | 5924 | L | 67 |  |  |  |
| cox1 |  | 5929 | 7479 | H | 1551 | ATA | AGG | +4 |
| trnS2 (tga) | Ser | 7471 | 7541 | L | 71 |  |  | –9 |
| trnD (gtc) | Asp | 7543 | 7611 | H | 69 |  |  | +1 |
| cox2 |  | 7612 | 8299 | H | 688 | ATG | T* |  |
| trnK (ttt) | Lys | 8300 | 8367 | H | 68 |  |  |  |
| trnK' (---) |  | 9097 | 9161 | H | 65 |  |  | +731 |
| atp8 |  | 9163 | 9327 | H | 164 | ATG | TAG | +1 |
| atp6 |  | 9324 | 10001 | H | 678 | ATA | TA* | –4 |
| cox3 |  | 10000 | 10785 | H | 786 | ATG | T* | –2 |
| trnG (tcc) | Gly | 10784 | 10852 | H | 69 |  |  | –2 |
| nad3 |  | 10853 | 11192 | H | 340 | ATG | T* |  |
| trnR (tcg) | Arg | 11193 | 11261 | H | 69 |  |  |  |
| nad4l |  | 11262 | 11546 | H | 285 | ATG | TAA |  |
| nad4 |  | 11537 | 12914 | H | 1377 | ATG | TAA | –10 |
| trnH (gtg) | His | 12900 | 12968 | H | 69 |  |  | –15 |
| trnS1 (gct) | Ser | 12969 | 13036 | H | 68 |  |  |  |
| nad5 |  | 13069 | 14860 | H | 1792 | ATG | T* | +34 |
| nad6 |  | 14858 | 15358 | L | 501 | ATG | AGA | –3 |
| trnE (ttc) | Glu | 15360 | 15428 | L | 69 |  |  | +29 |
| Cytb |  | 15431 | 16573 | H | 1125 | ATG | TAA | +70 |
| D-loop |  | 16574 | 16795 |  | 221 |  |  |  |

Note: rrnS and rrnL: 12S and 16S ribosomal RNAs; nad1–6 and 4l: NADH dehydrogenase subunits 1–6 and 4l; cox 1-3: cytochromec oxidase subunits 1-3; atp 6 and 8: ATP synthase subunits 6 and 8; Cytb: Cytochrome b; trn: transfer RNA (tRNA); bp, base pair(s).

*– Incomplete stop codons: complete stop codon would be generated by the post-transcriptional polyadenylation machinery.

#– spacer (+) and overlap (–).
