# Supplementary material for: Next-generation sequencing of mixed genomic DNA allows efficient assembly of rearranged mitochondrial genomes in Amolops chunganensis and Quasipaa boulengeri
Source: PeerJ. 2016 Dec 15;4:e2786. doi: 10.7717/peerj.2786 (PMC5162401; doi:10.7717/peerj.2786)
Supplement: Table S3 [file peerj-04-2786-s005.docx]

Table S3. Location of features in the mtDNA of *Quasipaa boulengeri*.

| Name | Amino acids | Position | | Stranad | Length | Codon | | Intergenic nucleotides # |
| --- | --- | --- | --- | --- | --- | --- | --- | --- |
|  |  | From | To |  |  | Start | Stop |  |
| D-loop |  | 1 | 394 |  | 394 |  |  |  |
| trnL1 (tag) | Leu | 395 | 466 | H | 72 |  |  |  |
| trnT (tgt) | Thr | 467 | 536 | H | 70 |  |  |  |
| trnP (tgg) | Pro | 537 | 605 | L | 69 |  |  |  |
| trnF (gaa) | Phe | 605 | 673 | H | 69 |  |  | –1 |
| rrnS |  | 674 | 1606 | H | 933 |  |  |  |
| trnV (tac) | Val | 1606 | 1673 | H | 68 |  |  | –1 |
| rrnL |  | 1674 | 3255 | H | 1582 |  |  |  |
| trnL2 (taa) | Leu | 3255 | 3327 | H | 73 |  |  | –1 |
| nad1 |  | 3331 | 4285 | H | 945 | ATG | T* | +3 |
| trnI (gat) | Ile | 4286 | 4356 | H | 71 |  |  |  |
| trnQ (ttg) | Gln | 4357 | 4427 | L | 71 |  |  |  |
| trnM (cat) | Met | 4427 | 4495 | H | 69 |  |  | –1 |
| trnM (cat) | Met | 4500 | 4568 | H | 69 |  |  | +4 |
| nad2 |  | 4569 | 5601 | H | 1032 | ATT | T* |  |
| trnW (tca) | Trp | 5602 | 5671 | H | 70 |  |  |  |
| trnA (tgc) | Ala | 5672 | 5740 | L | 69 |  |  | +2 |
| trnN (gtt) | Aln | 5743 | 5815 | L | 73 |  |  | +2 |
| Rep_Origin (O_L_) |  | 6105 | 6134 | L | 30 |  |  | +289 |
| trnC (gca) | Cys | 6392 | 6457 | L | 66 |  |  | +259 |
| trnY (gta) | Tyr | 6458 | 6524 | L | 67 |  |  |  |
| cox1 |  | 6529 | 8079 | H | 1551 | ATA | AGG | +4 |
| trnS2 (tga) | Ser | 8071 | 8141 | L | 71 |  |  | –9 |
| trnad (gtc) | Asp | 8142 | 8210 | H | 69 |  |  |  |
| cox2 |  | 8213 | 8897 | H | 684 | ATG | T* | +2 |
| trnK (ttt) | Lys | 8898 | 8968 | H | 71 |  |  |  |
| atp8 |  | 8970 | 9131 | H | 162 | ATG | TAA | +1 |
| atp6 |  | 9125 | 9808 | H | 684 | ATG | T* | –7 |
| cox3 |  | 9807 | 10590 | H | 784 | ATG | T* | –2 |
| trnG (tcc) | Gly | 10591 | 10659 | H | 69 |  |  |  |
| nad3 |  | 10660 | 10996 | H | 337 | ATG | T* |  |
| trnR (tcg) | Arg | 10997 | 11065 | H | 69 |  |  |  |
| nad4L |  | 11067 | 11351 | H | 285 | ATG | TAA | +1 |
| nad4 |  | 11345 | 12707 | H | 1363 | ATG | T* | –7 |
| trnH (gtg) | His | 12708 | 12776 | H | 69 |  |  |  |
| trnS1 (gct) | Ser | 12777 | 12844 | H | 68 |  |  |  |
| nad5 |  | 12890 | 14713 | H | 1823 | ATG | TAG | +45 |
| nad6 |  | 14699 | 15199 | L | 501 | ATG | AGG | –13 |
| trnE (ttc) | Glu | 15197 | 15265 | L | 69 |  |  | –1 |
| Cytb |  | 15273 | 16418 | H | 1146 | ATG | TAA | +7 |
| D-loop |  | 16419 | 16672 |  | 253 |  |  |  |

Note: rrnS and rrnL: 12S and 16S ribosomal RNAs; nad1–6 and 4l: NADH dehydrogenase subunits 1–6 and 4l; cox 1-3: cytochromec oxidase subunits 1-3I; atp 6 and 8: ATP synthase subunits 6 and 8; Cytb: Cytochrome b; trn: transfer RNA (tRNA); bp, base pair(s).

*– Incomplete stop codons: complete stop codon would be generated by the post-transcriptional polyadenylation machinery.

#– spacer (+) and overlap (–).
